# Understanding Internet Use Among Dementia Caregivers: Results of Secondary Data Analysis Using the US Caregiver Survey Data

**DOI:** 10.2196/ijmr.3127

**Published:** 2015-02-23

**Authors:** Heejung Kim

**Affiliations:** ^1^University of KansasSchool of NursingKasnas City, KSUnited States

**Keywords:** Internet, dementia, caregiver, stress, consumer health information

## Abstract

**Background:**

Informal caregivers of persons with dementia experience higher levels of chronic stress in the caregiving trajectory. The Internet provides diverse types of caregiver resources that may help ameliorate their stress and relevant negative outcomes. However, there is limited information about the prevalence and factors of using Internet-based resources for health- and caregiving-related purposes in informal caregivers of persons with dementia.

**Objective:**

Specific aims of this study were (1) to determine the prevalence and factors of caregiver’s health-related Internet use and (2) to compare sociodemographic and caregiving-related characteristics between health-related Internet users and non–health-related Internet users among informal caregivers of persons with dementia.

**Methods:**

This quantitative investigation was a descriptive correlational design using a secondary data analysis. Primary data were collected via a survey conducted in 2009 by the National Alliance for Caregiving and the American Association of Retired Persons. Telephone interviews utilizing standardized questionnaires were used to collect self-reported information about sociodemographics and caregiving-related history (N=450). Descriptive statistics and a hierarchical binary logistic regression analysis were completed based on the stress process model.

**Results:**

Approximately 59% (265/450) of dementia caregivers were identified as health-related Internet users. Caregivers’ sociodemographics and their subjective responses of caregiving stress were the most significant factors to identify health-related Internet users followed by workload assisting in instrumental activities of daily living of persons with dementia. There were significant differences for caregiver’s age, levels of education and income, hours spent caregiving, and the relationship to persons with dementia between health-related Internet users and non–health-related Internet users (*P*<.05 for all). After controlling for confounding effects, younger age of persons with dementia (OR 0.278, 95% CI 0.085-0.906), higher education levels of caregivers (OR 3.348, 95% CI 2.019-5.552), shorter caregiving time spent per week (OR 0.452, 95% CI 0.243-0.840), higher levels of caregiver’s emotional stress (OR 1.249, 95% CI 1.004-1.555), and financial hardship (OR 4.61, 95% CI 1.416-14.978) were identified as newly emerging factors of health-related Internet use.

**Conclusions:**

Although the Internet provided useful resources for caregivers of persons with dementia, dementia caregivers reported lower levels of health-related Internet use compared to the general public. Our findings confirmed the impact of age, education levels, and/or income on Internet use reported in previous studies. However, the predictive value of subjective responses of caregiving stress for health-related Internet use was a new addition. These findings will assist health care providers, researchers, and policy makers in identifying who is the least likely to access Internet-based resources and how Internet-based strategies can best be designed, implemented, and distributed to meet the needs of this group of users.

## Introduction

Dementia, including Alzheimer disease, refers to cognitive disorders presenting memory impairment, difficulty in language, organizational ability, abstract thinking, object recognition, and disturbance of executive function [1]. Adults caring for persons living with dementia (hereafter dementia caregivers) are the second-largest informal caregiver group in the United States because most people with dementia are older adults aged 65 or older [1,2]. Dementia caregivers are more likely to experience a wide range of negative behaviors or health problems than persons with nondementia health problems [2,3]. For example, dementia caregivers frequently exhibit maladaptive coping strategies, express concern about their poor quality of life, experience lower self-rated health, and report a higher level of caregiver burden [4-6]. In addition, dementia caregivers report severe sleep disturbances, clinical depression, and higher mortality compared to other caregivers [4,6-8]. Interestingly, these negative effects of dementia caregiving project to their care recipients because dyads of caregivers and persons with dementia are interdependent in the family unit [9]. Caregiver stress and burden have been shown to increase caregiver’s harmful or abusive behaviors toward their care recipients [10], accelerate the early placement of persons with dementia into institutional care [11], and decrease the life expectancy of care recipients [12]. Thus, timely reduction of caregiver stress and related problems are critical for both caregivers and care recipients.

To discontinue this vicious cycle between caregivers and care recipients, the stress process model emphasizes the proper use of resources to mediate the relationship between caregiver stress and relevant consequences [13]. Caregivers will experience higher levels of stress if they perceive their demands to be beyond the capacity of their coping resources [13]. Dementia caregivers are likely to seek out external resources that will help them resolve their stress, manage their health problems, and provide ongoing care for persons with dementia [2,3,12]. However, previous studies of resource use have generally focused on traditional face-to-face resources, such as professional health care services and support [6]; community-based services, such as respite services or caregiving assistance from professionals or nonprofessionals [14]; or agency-provided health and human resources [15]. However, there are no investigations examining Internet-based resources for health-related purposes in dementia caregivers.

Internet-based health resources include health information on websites and activities via communication technologies, whereas it excludes specific health interventions based on information and communication technology (ICT) specifically designed by clinical researchers [16,17]. Internet-based resources are now widely used and well integrated into the daily lives of the caregiving population [18]. The Internet modality assists in overcoming the limitations of a face-to-face approach; namely, time constraints, geographic limitations, and transportation issues [19,20]. Surveys of family caregivers report that 80% to 95% request technology-based interventions and Internet-based information or resources for enhancing better caregiving on behalf of their care recipients [21,22]. Clinicians have suggested various online information to caregivers as practical adjuncts or alternatives to traditional approaches without proven evidence [23]. However, it has rarely been evaluated in terms of how much dementia caregivers use the Internet for health-related purposes (hereafter health-related Internet use) and what factors affect their use. Thus, evidence-based practice requires more data to support current clinical practice.

This study used a modified stress process model by adding health-related Internet use as new subcomponent of resources. The original framework, developed by Pearlin et al [13] in 1990, has been used to understand how caring for a person with dementia affects both the health and well-being of both persons with dementia and their informal caregivers. In the multidimensional caregiving stress process, appropriate use of external resources mediates their coping within stressful situations. The addition of health-related Internet use to this original model can reflect the current need for virtual care resources for dementia caregivers living in the high-tech society of the 21st century [21,22]. The modified model was used for this study regarding (1) defining study constructs, (2) selecting and operationalizing study variables, (3) guiding data analysis, and (4) interpreting findings for clinical inferences.

This study evaluated how dementia caregivers use Internet-based resources for health- and caregiving-related purposes. Specifically, this study examined the prevalence and factors of health-related Internet use in dementia caregivers as well as differences between Internet users and non-Internet users. Three research questions were proposed:

1. What percentage of dementia caregivers use Internet-based resources for health- and caregiving-related purposes?
2. Are there any differences in sociodemographic and caregiving-related characteristics between health-related Internet users and non–health-related Internet users?
3. Which sociodemographic and caregiving-related factors are associated with health-related Internet use in dementia caregivers?

## Methods

### Design

The study was a cross-sectional and descriptive correlational design using a secondary data analysis. The primary data source was from the National Alliance for Caregiving (NAC) and the American Association of Retired Persons (AARP). This dataset was selected for this study because of (1) its up-to-date information on Internet use by dementia caregivers and (2) continuous refinement of sampling and data collection over the past decade [24,25].

### Description of the Primary Data Source

The NAC and AARP survey collected sociodemographic and caregiving-related data about persons with dementia and caregivers as well as their Internet and technology use. Interviews using a standardized questionnaire were programmed into a computerized telephone system and were conducted from March to June 2009. Interviewees were 6806 adults living in communities in California, Delaware, Illinois, Kansas, Ohio, Virginia, and the state of Washington in the United States. Random digit dialing based on surnames produced a set of telephone numbers stratified by geographic population density. Oversampling was done in racial and ethnic minorities (African American, Asian, and Hispanic groups) and older adults (age 50 years or older). One respondent was randomly selected from each household. If there were multiple care recipients (2 or more) for 1 caregiver, interviewers focused on the information for 1 primary care recipient who was receiving the most assistance from the caregiver [24,25].

### Sample

**Figure 1:**
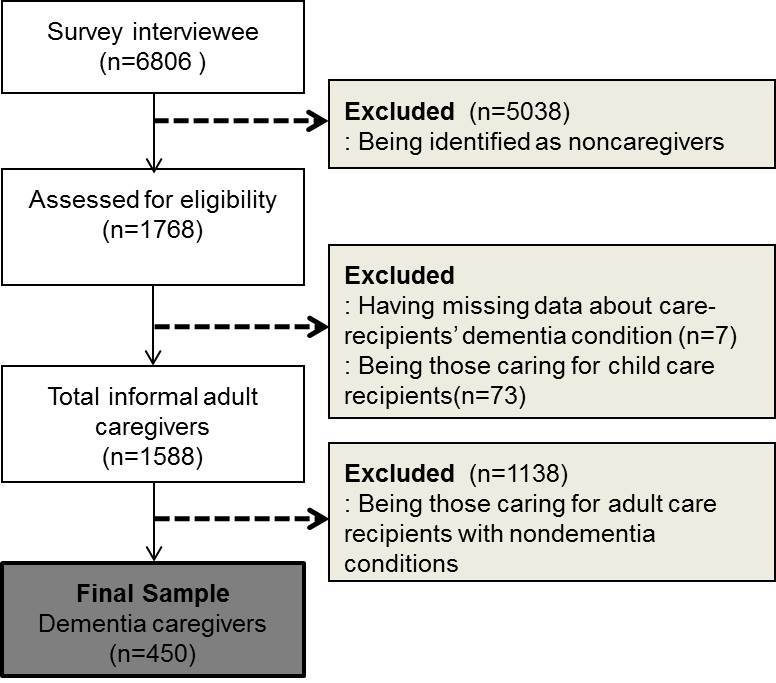
Flowchart of study samples.

This study used a subset of the data belonging to persons with dementia and their caregivers. Dementia caregivers were defined as persons who provided unpaid care or assistance to a family, relative, friend, or anyone living with Alzheimer dementia, any other type of dementia, or dementia-related conditions (confusion or forgetfulness). Among the 1768 informal caregivers in the dataset, 450 eligible caregivers were included in this study after excluding those caring for persons younger than 18 years (n*=*173), anyone with incomplete data regarding their care recipients’ dementia condition (n*=*7), and nondementia caregivers (n*=*1138) (See Figure 1).

### Measures

#### Baseline Information on Dementia Care Recipients and Their Caregivers

Both persons with dementia and their caregiver’s sociodemographic information were collected: age, gender, race/ethnicity, education level, residence area, and income, as well as the relationship between persons with dementia and their caregivers. Ages were a continuous variable, whereas all others were categorical variables. References (coded as 0) were those who were male, non-Hispanic white, had less than college-level education, rural residents, nonfamily or nonrelatives, and a household income of less than US $30,000 per year.

#### Caregiving-Related Information

Dementia caregivers self-reported the number of hours of caregiving per week, the duration of caregiving in years, and subjective responses of caregiving stress. The number of hours spent on caregiving tasks indicated how many hours they devoted to caregiving per week (range 1-168): 1 indicated they spent 1 hour or less per week and 168 indicated they engaged in full-time caregiving work. The duration of the caregiving indicated how long they had been performing the caregiver role. Here, 1 indicated that at the time of the survey they had either spent 1 year or less as a caregiver or that they only occasionally provided caregiving on an on-and-off basis. Higher values represented the approximate number of years they had been providing care.

Objective caregiving stressors were primarily based on a functional dependency of persons with dementia in terms of activities of daily living (ADLs) and instrumental activities of daily living (IADLs). Here, the ADLs consisted of 6 activities: transferring, dressing, toileting, bathing, feeding, and handling incontinence or diapers (range 0-6) [26]. The IADLs were selected based on Lawton and Brody’s scale (range 0-6) [27] and included 6 activities: managing medications, managing finances, shopping, doing housework including laundry, preparing meals, and transportation. Higher scores indicated that persons with dementia were more dependent on caregivers in their daily living and for instrumental functions. In this study sample, Cronbach alpha of ADLs was .82 and the IADLs was .70. Subjective responses of caregiving stress were evaluated in terms of physical strain, emotional stress, and financial hardship. Each item was scored on a 5-point Likert scale, with higher scores indicating more physical strain, emotional stress, and financial hardship experienced. Moderate correlations were observed across the 3 items.

#### Health-Related Internet Use

The main focus of interest for this study was health-related Internet use, measured by self-reports of the frequency of Internet use for health-related purposes. Frequency of health-related Internet use was measured by asking the question, “How often, if at all, have you gone to Internet websites in the past year to find information and resources in any way related to being a caregiver for your care recipient? Often, sometimes, rarely, or never?” [16] This study used a consistent definition of health-related Internet use, which has been used in previous studies to compare prevalence [16,17]. Non-health-related Internet users were defined as those who never used Internet-based resources for health and caregiving purposes. All others were defined as health-related Internet users.

### Procedures

The data acquisition and use was approved by the NAC and AARP. All data provided were deidentified to follow Health Insurance Portability and Accountability Act privacy rules. The University of Virginia’s Institutional Review Board for Social and Behavioral Sciences reviewed this project and confirmed the exempt status. Data analyses included both preliminary analyses (reliability tests, intercorrelation analyses, an exploratory factor analyses, and relevant statistical assumptions checks) and main analyses (descriptive analyses and a hierarchical binary logistic regression analysis). A total of 13 cases were dropped from the main analysis based on listwise deletion across all independent and dependent variables. This represented only 2.9% of the sample; thus, no data imputation was conducted [28].

Before the descriptive and regression analysis, all statistical assumptions were checked including univariate/multivariate normality, linearity, and multicollinearity. To correct for univariate normality, the variable of age of persons with dementia, number of hours for caregiving, and financial hardship was transformed using a log_10_ function. For multivariate normality, 2 outliers were identified based on the Mahalanobis distance function. When comparing results from a model with 2 outliers to those from the model without the outliers, there were no differences in *R* ^2^, coefficients, *F* statistics, or *P* values, although the values of the Mahalanobis distance were corrected. To glean the maximum information from the available samples, the final results were reported from the model that did not exclude those 2 outliers. The final sample size (N*=*437) in the main analysis was sufficient for conducting multiple regression with 16 independent variables because the suggested sample size was 160-320 [28-30].

### Statistical Analysis

To answer the first and second research questions, percentage responding weighted frequency and means (SD) were reported as well as results of univariate descriptive statistics (independent *t* tests, Mann-Whitney *U* tests, or chi-square tests) to compare health-related Internet users to non-health-related Internet users in the dementia caregivers. To answer the third research question, a hierarchical binary logistic regression analysis was completed to identify factors of the health-related Internet user group (0=non-health-related Internet users; 1=health-related Internet users). Based on the stress process model [13], 16 independent variables were entered into the regression model. Block 1 included age and gender of persons with dementia, whereas Block 2 included sociodemographics of caregivers, including age, gender, education level, income, race and ethnicity, resident care, and relationship to persons with dementia. Separately, functional dependency in terms of ADLs and IADLs was included in Block 3 and caregiving history was included in Block 4. Subjective responses of caregiving stress were included in Block 5. SPSS 20.0 (IBM Corp, Armonk, NY, USA) was used for data analyses. The significance-level criterion for all statistical tests was alpha=.05, 2-tailed. To infer generalizable findings, the study applied a composite score for the population weight, which was calculated based on age, gender, and race/ethnicity, and the results compared to the 2008 population estimates released by the Population Division of the US Census Bureau on May 14, 2009 [24].

## Results

### Sample Characteristics of Persons With Dementia and Their Caregivers

The mean age of persons with dementia was 78.37 years (SD 14.13) and the majority were women (69.1%, 309/447). They had moderate levels of functional impairments of ADLs and IADLs (mean 2.21, SD 2.01 and mean 4.21, SD 1.71, respectively). The mean age of their caregivers was 50.30 years (SD 14.98). The majority of them were women (62.0%, 277/447), non-Hispanic whites (71.6%, 320/450), and children or grandchildren of persons with dementia (74.9%, 335/447). Caregivers were educated at the level of high school or less (53.5%, 240/449) and had overall household incomes greater than US $30,000 per year (70.3%, 314/447). The residence areas of the caregivers were evenly distributed (urban: 29.3%, 131/447; suburban: 37.8%, 169/447; rural: 30.7%, 137/447). Caregivers spent a mean 29.96 (SD 46.93) hours per week for a mean of 5.22 (SD 7.96) years performing the role of caregiver for the person with dementia.

### Comparison of Health-Related and Non–Health-Related Internet Users

Approximately 59% (265/450, 58.9%) of dementia caregivers were identified as health-related Internet users. Several of the caregiver’s characteristics were statistically different between health-related Internet users and non–health-related Internet users. Health-related Internet users were younger (*P*=.01), were more educated (*P*<.001), had a higher level of household income (*P*<.001), and spent fewer hours per week caregiving (*P*=.004). Health-related Internet users were more likely to be a child or grandchild of persons with dementia (78.4%, 207/265) rather than their spouse (3.4%, 9/265) compared to non–health-related Internet users (child or grandchild: 70.0%, 128/185; spouses: 10.9%, 20/185; *P*=.02). However, the sociodemographic characteristics of persons with dementia were not statistically different whether their caregivers were health-related Internet users or not (Table 1).

### Overall Model of a Hierarchical Binary Logistic Regression

The results of the hierarchical binary logistic regression analysis are shown in Table 2. The overall model explained 23.9% of the variance to predict who health-related Internet users were. The group classifications predicted 80.3% of the health-related Internet user group and 55.4% of the non–health-related Internet user group (Tables 2 and 3).

In Block 1, the age and gender of persons with dementia explained 1.3% of the variance (Nagelkerke *R* ^2^=.013; χ^2^_2_=3.7; *P=*.15). Both the age and gender of persons with dementia were not significant factors. After adding caregiver’s sociodemographic factors into Block 2, the overall model became significant because Block 2 added 16.9% of the explained variance (Nagelkerke *R* ^2^=.182; χ^2^_15_=54.4; *P*<.001). Caregiver’s age (Wald χ^2^_1_=4.5; *P=*.03; OR 0.980, 95% CI 0.963-0.998) and education levels (Wald χ^2^_1_=25.8; *P*<.001; OR 3.523, 95% CI 2.168-5.726) were significant factors. Block 3 including ADLs and IADLs did not significantly increase explained variance (Nagelkerke *R* ^2^=.003; χ^2^_2_=0.8; *P=*.67). However, overall model and predictive values of caregiver’s age and education levels still remained significant (*P*<.05 for all). Block 4 included the number of hours and duration of caregiving years which did not significantly increase explained variance (Nagelkerke *R* ^2^=.016; χ^2^_2_=5.1; *P=*.08). However, the overall model including Blocks 1 to 4 still remained significant (Nagelkerke *R* ^2^=.201; χ^2^_19_=60.3; *P*<.001). There were noticeable changes in individual factors. Caregiver’s age became an insignificant factor (Wald χ^2^_1_=2.5; *P=*.11). After controlling for sociodemographics of persons with dementia and their caregivers, IADLs became a significant factor (Wald χ^2^_1_=4.0; *P=*.045; OR 1.201, 95% CI 1.004-1.436). After controlling for functional dependency of persons with dementia and all sociodemographics, the number of hours for caregiving was a significant factor (Wald χ^2^_1_=4.8; *P=*.03; OR 0.519, 95% CI 0.288-0.933). However, caregiver’s education levels remained a factor with similar predictive strength (Wald χ^2^_1_=25.1; *P*<.001; OR 3.536, 95% CI 2.158-5.794).

The final model including Blocks 1 to 5 significantly explained 23.9% of the variance (Nagelkerke *R* ^2^=.239; χ^2^_22_=73.0; *P*<.001) with Block 5′s significant increase of 2.0% of explained variance (χ^2^_3_=12.7; *P=*.01). Caregiver’s age still remained an insignificant factor (Wald χ^2^_1_=3.4; *P=*.07). However, caregiver’s education levels remained a factor with similar predictive strength (Wald χ^2^_1_=21.9; *P*<.001; OR 3.348, 95% CI 2.0-5.552). After controlling for sociodemographics of persons with dementia and their caregivers, IADLs became an insignificant factor (Wald χ^2^_1_=3.2; *P=*.07; OR 1.183, 95% CI 0.985-1.420). After controlling for functional dependency of persons with dementia and all sociodemographic factors, the number of hours for caregiving remained a significant factor, but decreasing in strength of prediction (Wald χ^2^_1_=6.3; *P=*.01; OR 0.452, 95% CI 0.243-0.840). Newly emerging significant factors were identified. Age of persons with dementia was shown as a significant factor (Wald χ^2^_1_=4.5; *P=*.03; OR 0.278, 95% CI 0.085-0.906). Caregiver’s emotional stress (Wald χ^2^_1_=4.0; *P=*.046; OR 1.249, 95% CI 1.004-1.555) and caregiver’s financial hardship (Wald χ^2^_1_=6.4; *P=*.01; OR 4.606, 95% CI 1.416-14.978) were significant factors after controlling for sociodemographics, caregiving history, and functional dependency of persons with dementia.

**Table 1:** Sociodemographic and caregiving-related characteristics of persons with dementia and their caregivers.

Variables			All N=450	Health-related Internet users (n=265)	Non–health-related Internet users (n=185)	*P*
**Description of persons with dementia** ^a^						
	Age (years), mean (SD)^b^		78.37 (14.1)	79.38 (12.8)	76.90 (15.8)	.07
	Impairment of ADLs, mean (SD)^c^		2.21 (2.0)	2.14 (2.1)	2.32 (1.9)	.36
	Impairment of IADLs, mean (SD)^c^		4.21 (1.7)	4.22 (1.7)	4.18 (1.8)	.80
	Gender (female), n (%)^d^		309 (69.1)	180 (68.2)	129 (70.5)	.60
**Description of their caregivers**						
	Age (years), mean (SD)^c^		50.30 (15.0)	48.67 (13.3)	52.62 (16.8)	.009
	Number of hours for caregiving (per week), mean (SD)^b^		29.96 (46.9)	26.10 (44.3)	35.57 (50.1)	.004
	Duration of caregiving (years), mean (SD)^b^		5.22 (8.0)	4.65 (6.6)	6.05 (9.6)	.35
	Gender (female), n (%)^d^		277 (62.0)	156 (58.9)	121 (66.1)	.12
	**Race, n (%)** ^d^					.28
		Non-Hispanic white	320 (71.6)	195 (73.9)	125 (68.3)	
		Non-Hispanic African American	49 (11.0)	24 (9.1)	25 (13.7)	
		Hispanic	53 (11.9)	27 (10.2)	26 (14.2)	
		Non-Hispanic Asian	12 (2.7)	7 (2.7)	5 (2.7)	
		Missing data	16 (2.8)	12 (4.1)	4 (1.1)	
	**Education levels, n (%)** ^d^					<.001
		High school or less	240 (53.5)	109 (40.9)	131 (71.6)	
		Some college or higher	208 (46.5)	156 (59.1)	52 (28.4)	
	**Residence area, n (%)** ^d^					.05
		Urban	131 (29.3)	82 (31.1)	49 (26.8)	
		Suburban	169 (37.8)	108 (40.9)	61 (33.3)	
		Rural	137 (30.7)	70 (26.5)	67 (36.6)	
		Missing data	13 (2.2)	5 (1.5)	8 (3.3)	
	**Income (US $), n (%)** ^d^					<.001
		<$30,000/year	94 (21.0)	39 (14.8)	55 (30.1)	
		≥$30,000/year	314 (70.3)	201 (76.1)	113 (61.8)	
		Missing data	42 (8.7)	25 (9.1)	17 (8.1)	
	**Relationship to person with dementia, n (%)** ^d^					.02
		Spouse	29 (6.5)	9 (3.4)	20 (10.9)	
		Parent	15 (3.4)	7 (2.7)	8 (4.3)	
		Child or grandchild	335 (74.9)	207 (78.4)	128 (70.0)	
		Other type of relative	38 (8.5)	22 (8.3)	16 (8.6)	
		Friend/nonrelative/neighbor	29 (6.5)	18 (6.8)	11 (6.0)	
		Missing data	4 (0.2)	2 (0.4)	2 (0.2)	
	**Subjective responses of caregiving stress**					
		Physical strain, mean (SD)^c^	2.42 (1.4)	2.36 (1.3)	2.51 (1.5)	.27
		Emotional stress, mean (SD)^c^	3.13 (1.3)	3.20 (1.3)	3.03 (1.3)	.19
		Financial hardship, mean (SD)^b^	2.05 (1.3)	2.08 (1.3)	2.01 (1.3)	.51

^a^ ADL=activities of daily living; IADL=instrumental activities of daily living.

^b^ Tested by Mann-Whitney *U* tests.

^c^ Tested by independent *t* test.

^d^ Tested by chi-square test.

**Table 2:** Final model of a hierarchical binary logistic regression analysis to predict health-related Internet users.

Factors		B (SE)	Wald χ^2^ (*df*)	*P*	OR (95% CI)
**Block 1: Demographics of persons with dementia**					
	Constant	1.461 (1.320)	1.2 (1)	.27	4.309
	Age^a^	–1.279 (0.602)	4.5 (1)	.03	0.278 (0.085-0.906)
	Female gender^b^	0.102 (0.276)	0.1 (1)	.71	1.108 (0.646-1.901)
**Block 2: Sociodemographics of caregivers**					
	Age	–0.019 (0.010)	3.7 (1)	.07	0.981 (0.962-1.001)
	Female gender^b^	–0.365 (0.250)	2.1 (1)	.15	0.694 (0.425-1.134)
	Education levels^c^	1.208 (0.258)	21.9 (1)	<.001	3.348 (2.019-5.552)
	Household income^d^	0.593 (0.318)	3.5 (1)	.06	1.809 (0.969-3.377)
	Race and ethnicity		0.6 (3)	.89	
	Resident area		1.7 (2)	.42	
	Relationship to dementia persons		2.8 (4)	.59	
**Block 3: Functional dependency**					
	ADLs	–0.010 (0.070)	0.02 (1)	.88	0.990 (0.862-1.136)
	IADLs	0.168 (0.093)	3.2 (1)	.07	1.183 (0.985-1.420)
**Block 4: Caregiving history**					
	Number of hours for caregiving^a^	–0.795 (0.317)	6.3 (1)	.01	0.452 (0.243-0.840)
	Duration of caregiving	–0.023 (0.020)	1.2 (1)	.27	0.978 (0.939-1.017)
**Block 5: Subjective responses of caregiving stress**					
	Physical strain	–0.100 (0.117)	0.7 (1)	.39	0.905 (0.719-1.138)
	Emotional stress	0.222 (0.112)	4.0 (1)	.05	1.249 (1.004-1.555)
	Financial hardship^a^	1.527 (0.602)	6.4 (1)	.01	4.606 (1.416-14.978)

^a^ Transformed using a log_10_ function.

^b^ Reference: male.

^c^ Reference: those educated at the level of high school or less.

^d^ Reference: those who had household incomes less than US $30,000 per year.

**Table 3:** Odds ratio changes of significant factors.

Factors		Block 1		Block 1 and 2		Block 1 to 3		Block 1 to 4		Block 1 to 5	
		OR	*P*	OR	*P*	OR	*P*	OR	*P*	OR	*P*
Constant		4.84	.13	4.77	.20	4.80	.21	3.07	.38	4.31	.27
**Block 1: Demographics of persons with dementia**											
	Age^a^									0.28	.03
	Female gender^b^										
**Block 2: Sociodemographics of caregivers**											
	Age	—		0.98	.17	0.98	.02	0.99	.13	0.98	.07
	Female gender^b^	—									
	Education levels^c^	—		3.52	<.001	3.54	<.001	3.54	<.001	3.35	<.001
	Household income^d^	—									
	Race and ethnicity	—									
	Resident area	—									
	Relationship with dementia persons	—									
**Block 3: Functional dependency**											
	ADLs	—		—							
	IADLs	—		—				1.20	.04	1.18	.07
**Block 4: Caregiving history**											
	Number of hours for caregiving^a^	—		—		—		0.52	.03	0.45	.01
	Duration of caregiving	—		—		—					
**Block 5: Subjective responses of caregiving stress**											
	Physical strain	—		—		—		—			
	Emotional stress	—		—		—		—		1.25	.04
	Financial hardship^a^	—		—		—		—		4.61	.01

^a^ Transformed using a log_10_ function.

^b^ Reference: male.

^c^ Reference: those educated at the level of high school or less.

^d^ Reference: those who had household incomes less than US $30,000 per year.

## Discussion

### Principal Results

This study examined the sociodemographic and caregiving characteristics of health-related Internet users among dementia caregivers. In this study, using 2009 NAC and AARP survey data, 59.1% of dementia caregivers were identified as health-related Internet users. Caregiver’s age, levels of education and income, hours spent caregiving each week, and relationship to persons with dementia were univariate factors discriminating the health-related Internet use group from non-health-related users. After controlling for confounding effects, age and dependency of IADLs of persons with dementia, caregiver’s emotional stress, and caregiver’s financial hardship were newly emerging factors of health-related Internet use. Caregiver’s sociodemographics and their subjective responses of caregiving stress were the most significant factors to identify health-related Internet users followed by workload assisting in IADLs of persons with dementia [31].

### Comparison With Prior Work

In all, 59% of the prevalence of health-related Internet use is lower than that of the general public (80% in the 2010 Pew Internet & American Life Project) [21], but similar to those found in other types of caregivers (eg, 42%-60% for cancer caregivers) [17]. Compared to findings reported in the recent Pew Internet Health Tracking Survey (2012), our dementia caregiving participants reported a much higher rate (59%) than 39% reported by general caregivers of an adult or child with significant health issues [32]. This finding suggests that using the Internet has become more prevalent and significant behavior seeking for health-related resources among caregivers in the United States. This intensity seems to result from (1) the huge growth in availability and the widespread adoption of the Internet and relevant technologies [33], (2) health care consumers’ strong motivation, (3) their positive perceptions regarding Internet-based resources [34], and (4) the promising benefits of Internet-based approaches (ie, convenience and confidentiality) [34]. Thus, the Internet has been acknowledged as a promising modality for implementing interventions or distributing caregiving resources.

Caregiver’s sociodemographic characteristics are strong factors in determining their behavior regarding health-related Internet use, including age, education levels, income, and their relationship to persons with dementia. Similar to previous study findings, the predictive values of age and education levels were confirmed in this study. Younger and more highly educated caregivers reported they used Internet-based resources for better health and caregiving purposes similar to the same findings in the general population or of cancer caregivers [17,33-36]. Interestingly, those who had a minimum of college-level education were 3.35 times more likely to be health-related Internet users than those who were educated at the level of high school or less. Age and educational attainment were the most significant factors for eHealth literacy [37,38]. In addition, younger generations were generally considered to be more technology-friendly and more prepared to use Internet-based resources compared to their older counterparts. Additionally, a higher education level may be associated with either a higher level of knowledge of health-related resources or better computer skills [15,38].

Higher levels of both household income and self-reported financial hardship were associated with a greater likelihood of being health-related Internet users. Initially, those 2 findings may appear to conflict with one other because a basic assumption is that a person with higher income would experience a lower level of self-reported financial hardship. However, this inconsistency has been reported in previous research; as with the study findings, individuals with higher levels of income use the Internet more [33,35]. Individuals with higher household incomes are more likely to own computers and handheld mobile devices and spend more time using the Internet in their daily life than their lower-income counterparts [33,37]. However, another study has described the opposite association between income and health-related Internet use. A study of national surveys reported that those with lower incomes were also more likely to participate in online support groups than those with higher incomes [36]. Interestingly, this study found the unique impact of the subjective response of financial hardship after controlling for income levels. One possible explanation is that individuals with higher incomes may have diverse means to reduce their stress levels. However, those who are experiencing financial hardship have limited use of traditional face-to-face resources for stress reduction and they try to find alternatives through the Internet [33].

Spousal caregivers were significantly less likely to use available services, which is consistent with previous reports [14]. This reluctance by spousal caregivers seems to originate from emotional barriers: spouses tend to perform the caregiving role without external help because using resources may be considered as betraying the spousal relationship [14]. However, the significant impact of their relationship seems to result from the confounding effect of their age. After controlling for their age, the predictive value is no longer significant in this study sample. Additionally, children of persons with dementia would naturally be considerably younger than their parent and, thus, likely to be more technology-prepared [39].

One of the salient findings of this study was that the higher the emotional stress experienced by dementia caregivers, the more health-related Internet use they reported. When they reported very much emotional stress (score 5) on a 5-point Likert-type scale, they were 3.05 times more likely to be health-related Internet users than those who reported no emotional stress (score 1). This tendency was found in a previous study that applied qualitative methods to the online postings of dementia caregivers. The emotional concern and seeking psychosocial support represents the second-commonest theme for dementia caregivers after behavior of seeking information [20]. Caregiver stress has been shown as a need variable that facilitates their use of resources [40], especially in dementia caregivers [6,15,40]. Dementia caregivers with higher levels of emotional and psychological stress are more likely to use the traditional face-to-face resources of health and human services [15]. The stress-appraisal theory [41] and the stress process model [13] explain that there is a positive relationship between recognized stress levels and efforts to alleviate stress. In addition, a systemic review of networked technologies in dementia caregiver stated a potential impact of ITC intervention on caregiver stress [42]. Thus, health-related Internet use may be considered a coping strategy for caregivers to relieve their subjective stress or burden [41] and a mediator to modulate the impact of caregiving stress on negative outcomes [13].

The predictive value of functional dependency of IADLs appears after controlling for sociodemographic impact. When caregivers are taking care of totally dependent persons with dementia (IADLs score=6), they were 2.99 times less likely to be health-related Internet users than those taking care of totally independent persons with dementia (IALDs score=0). However, when adding the number of hours for caregiving, the predictive value of IADLs dependency became insignificant. The fewer hours caregivers spent caregiving, the more likely they were to be health-related Internet users. For those caring for cancer survivors, having fewer direct caring responsibilities increases the likelihood of health-related Internet use [17]. When caregivers spent longer times providing direct caregiving tasks, they did not have the time or energy to search for information and seek support via the Internet. Respite care may be especially beneficial to caregivers because it may free up the time they need to access the Internet for health-related purposes. Thus, this finding may suggest more appropriate ways to apply interventions designed for providing physical assistance and, thereby, reducing the workload of dementia caregivers.

### Study Limitations and Directions for Future Research

This study had several limitations: (1) limited inference of causality due to the cross-sectional design, (2) limited generalizability due to the use of a convenience sampling method, (3) possible responder bias in the self-reported data, and (4) difficulties in controlling data quality as a secondary data analysis.

Conceptually, dichotomous grouping (health-related Internet users vs non–health-related Internet users) has limitations in explaining their complex behavior of seeking resources for caregiving. Using current data, the multiple grouping depending on intensity of use (eg, high vs moderate vs nonuser groups) and multinomial logistic regression may show a more detailed description in this sample. Moreover, data collected in 2009 limitedly reflect current trends in the scientific community or daily practice because Internet research is 1 of the most rapidly changing in the field.

Future up-to-date studies would greatly benefit from the use of a longitudinal design that utilizes other multiple measurements of health-related Internet use or caregiver stress. Because the variables tested in this study are predisposing and need variables to facilitate health care service use [14], the inclusion of enabling variables related to health service utilization should provide a contextually better in-depth understanding to shed new light on the complex picture of health-related Internet use in this population. To define the medical condition of dementia using International Classification of Diseases codes will be more accurate than caregiver-reported condition.

### Conclusions

The Internet has become a significant resource for dementia caregivers for health-related purposes. This research adds to our knowledge of the prevalence and factors of health-related Internet use by dementia caregivers. Subjective responses of caregiving stress are a need factor leading to increased Internet use for health and caregiving purposes. Significant demographic factors provide helpful information to identify those who are less likely to use Internet-based resources. The lowest utilization is detected in those who were older, a spouse, less educated, with lower incomes, and devoting longer times to caregiving. Thus, this study helps us identify underserved groups regarding virtual health care resources. Clinical researchers should consider our findings to develop tailored interventions and effective care delivery approaches targeting the virtually underserved caregiving population. Additionally, the study findings may assist policy makers seeking to distribute information, resources, and services via the Internet to help dementia caregivers and their care recipients with dementia.

## Abbreviations

AARP: American Association of Retired Persons
ADLs: activities of daily living
IADLs: instrumental activities of daily living
ICT: information and communication technology
NAC: National Alliance for Caregiving
